# On estimating the time to statistical cure

**DOI:** 10.1186/s12874-020-00946-8

**Published:** 2020-03-26

**Authors:** Lasse H. Jakobsen, Therese M.-L. Andersson, Jorne L. Biccler, Laurids Ø. Poulsen, Marianne T. Severinsen, Tarec C. El-Galaly, Martin Bøgsted

**Affiliations:** 1 Department of Clinical Medicine, Aalborg University, Sdr. Skovvej 15, Aalborg, 9000 Denmark; 2 Department of Hematology, Aalborg University Hospital, Sdr. Skovvej 15, Aalborg, 9000 Denmark; 3 Department of Medical Epidemiology and Biostatistics, Karolinska Institutet, Nobels Väg, Stockholm, 171 65 Sweden; 4 Department of Oncology, Aalborg University Hospital, Hobrovej 18-22, Aalborg, 9000 Denmark

**Keywords:** Statistical cure, Cure point, Cancer survival

## Abstract

**Background:**

The mortality risk among cancer patients measured from the time of diagnosis is often elevated in comparison to the general population. However, for some cancer types, the patient mortality risk will over time reach the same level as the general population mortality risk. The time point at which the mortality risk reaches the same level as the general population is called the *cure point* and is of great interest to patients, clinicians, and health care planners. In previous studies, estimation of the cure point has been handled in an ad hoc fashion, often without considerations about margins of clinical relevance.

**Methods:**

We review existing methods for estimating the cure point and discuss new clinically relevant measures for quantifying the mortality difference between cancer patients and the general population, which can be used for cure point estimation. The performance of the methods is assessed in a simulation study and the methods are illustrated on survival data from Danish colon cancer patients.

**Results:**

The simulations revealed that the bias of the estimated cure point depends on the measure chosen for quantifying the excess mortality, the chosen margin of clinical relevance, and the applied estimation procedure. These choices are interdependent as the choice of mortality measure depends both on the ability to define a margin of clinical relevance and the ability to accurately compute the mortality measure. The analysis of cancer survival data demonstrates the importance of considering the confidence interval of the estimated cure point, as these may be wide in some scenarios limiting the applicability of the estimated cure point.

**Conclusions:**

Although cure points are appealing in a clinical context and has widespread applicability, estimation remains a difficult task. The estimation relies on a number of choices, each associated with pitfalls that the practitioner should be aware of.

## Background

One of the most important aims of cancer patients is to become cured, which in general is different from reaching complete remission due to the risk of relapse, lethal side effects from the treatment, and late toxicities. However, for some cancer survivors the mortality risk reaches the same level as the general population mortality risk. This suggests a possible formulation of cure, namely *statistical cure*, which is achieved if the patients survive until the point at which the patient and general population mortality risks become similar [1]. The time point at which the patients become statistically cured is termed the *cure point* and its estimation is the main focus of this paper.

The problem of estimating cure points may be generalized by considering when the risk of a certain event in exposed individuals equals that of unexposed individuals. One example is smoking cessation, which is associated with a short term increased risk of developing type 2 diabetes, that over time gradually approaches that of non-smokers [2]. The ex-smoker may be interest in knowing when this risk is "normalized". Another example involves the fertility of women who have terminated the use of oral contraceptives, which is typically inferior to that of women who exclusively used diaphragms [3, 4]. However, as time progresses, the fertility returns to the level of previous diaphragm users. In this case the time at which the fertility level equals that of the control group may be of interest. In the present article, we only consider estimation of survival-related cure points for cancer patients, although the statistical problem applies to other diseases and endpoints.

Let *h* and *h*^∗^ be the hazard function of the patients and the general population, respectively. Inspired by Rabinowitz and Ryan [5], the cure point could be defined as

1 $$ t_{\epsilon} = \inf\left\{t | \text{ for all}\ s \geq t, h(s) - h^{*}(s) \leq \epsilon \right\},   $$

where *ε* is some margin of clinical relevance (MOCR), e.g., 0. Similarly to previous studies focusing on cure point estimation, the cure point could be estimated by sequentially testing for a difference between the patient and general population mortality (if *ε*=0) [6–8]. The cure point estimate is then the time point from which a significant difference is no longer observed. A similar time-varying test-statistic was established by Rabinowitz and Ryan [5]. However, as discussed by Rabinowitz and Ryan, with this approach it cannot be determined whether statistical cure has actually occurred or if there is simply not enough data to show a significant excess risk. As a consequence, with larger sample size, the cure point estimate will likely increase. The estimate can therefore only be considered a lower confidence bound for the cure point, which is difficult to interpret in a clinical context. Thus, approaches that directly involve hypothesis testing are not appropriate for cure point estimation.

Estimation of cure points without relying on classical hypothesis testing has previously been carried out in both applied and methodological studies [8–14]. In this article, we review and discuss existing methods and introduce two new approaches for cure point estimation from cancer survival data. The performance of the discussed methods is evaluated in a simulation study and we illustrate the methods on survival data from Danish colon cancer patients.

## Methods

Let *T* be the random survival time of each patient and *D* the random cause of death variable, which is either *cancer* or *other causes*. We denote the hazard and survival function of a patient with covariate vector z at time *t* by *h*(*t*|z) and *S*(*t*|z), respectively, and those of the general population, typically matched on variables such age, gender, and calendar year, by *h*^∗^(*t*|z) and *S*^∗^(*t*|z), respectively. To avoid basing the cure point estimation on hypothesis testing, researchers have pursued approaches based on a dynamic evaluation of the difference between the patient and general population mortality. Dynamic measures are used to provide conditional risks or rates given a certain history. For instance, it may be of interest to quantify the patient survival given that a patient has survived 2 years following the cancer diagnosis. The difference between the patient and general population mortality can be quantified by various measures, e.g., the excess hazard function, relative survival, or the loss in expectation of life [15]. In the present article, we term measures that dynamically quantify the difference in patient and general population mortality *comparison measures* and we estimate the cure point as the time point at which the applied comparison measure reaches some MOCR. Thus, in order to carry out cure point estimation, the investigator has to choose:

- A comparison measure, *G*(*t*|z), which dynamically quantifies the difference between the patient and general population mortality.
- An estimation procedure to compute the comparison measure.
- A MOCR for the comparison measure.

These choices are closely related since the choice of comparison measure should be related to both the possibility to define a MOCR, which is associated with its interpretation, and the ability to accurately compute the comparison measure. In the following, we describe different comparison measures, their interpretation, and methods to compute them. In particular, we describe the conditional relative survival [11], the relative survival of the uncured [9], and the probability of cure [13], which have previously been used for cure point estimation and introduce two new measures for estimating cure points, namely the probability of cancer-related death [16] and the loss of lifetime [17] (see Table 1 for an overview).

**Table 1:** Overview of comparison measures dynamically quantifying the difference between the patient and general population mortality

Comparison measure	Notation	Interpretation	Computability	Reference
Conditional relative survival	$\frac {R(t + u|\mathrm {z})}{R(t|\mathrm {z})}$	Challenging	Feasible	[11]
Relative survival of the uncured	*S*_*u*_(*t*|z)	Challenging	Challenging	[9]
Probability of cure	P(*Y*=1|*T*>*t*,z)	Intuitive	Challenging	[13]
Probability of cancer-related death	P(*T*≤*t*,*D*=cancer|z)	Intuitive	Challenging	[16]
Loss of lifetime	$\frac {\int _{t}^{\infty } S^{*}(u|\mathrm {z})du}{S^{*}(t|\mathrm {z})} - \frac {\int _{t}^{\infty } S(u|\mathrm {z})du}{S(t|\mathrm {z})}$	Intuitive	Challenging	[17]

### Previously used dynamic measures of excess mortality

#### Conditional relative survival

The relative survival function, *R*(*t*|z), is the ratio of the all-cause survival to the expected survival, i.e., *R*(*t*|z)=*S*(*t*|z)/*S*^∗^(*t*|z). Under some assumptions [18], the relative survival can be interpreted as the net survival, i.e., the survival of patients with a particular disease in the hypothetical scenario, where only deaths due to the disease are possible. However, these assumptions are usually not identifiable from the data and hence can be hard to justify [19].

Dal Maso et al. [11] estimated the cure point as the time at which the conditional relative survival,

2 $$ G(t|\mathrm{z}) = \frac{R(t + u|\mathrm{z})}{R(t|\mathrm{z})},  $$

was sufficiently high (e.g., >95%) for a certain time window, e.g., *u*=5 years.

However, determining a MOCR for the conditional relative survival requires in depth understanding of its meaning and scale. This can be challenging if the relative survival is interpreted as the net survival, because it reflects the survival in a hypothetical scenario. Even if the relative survival is not given a net survival interpretation, it remains a relative measure for which a MOCR is difficult to define because it neglects the absolute difference in mortality risk. Thus, the conditional relative survival may not be appropriate for cure point estimation. However, this approach is computationally feasible as various estimators for the relative survival exist [18, 20].

#### Cure models

Approaches based on functional parts of cure models have previously been proposed for cure point estimation. Cure models are particularly useful in scenarios with a plateau in the relative survival, which indicates that the patient mortality risk reaches the same level as the general population mortality risk [9]. Given some covariates, the main objective in cure models is to estimate the level at which the relative survival plateaus, also known as the *cure proportion*, which we denote by *π*(z). This is commonly done by formulating the relative survival as

3 $$ R(t|\mathrm{z}) = \pi(\mathrm{z}) + [1 - \pi(\mathrm{z})] S_{u}(t|\mathrm{z}).   $$

If the relative survival is interpreted as the net survival, *π*(z) is simply the probability of never experiencing a disease-related death [21]. This suggest that some individuals will be "cured" of the disease and hence never experience a disease-related death while others are uncured and eventually will die from the disease. Thus, the patient population is considered a mixture of cured and uncured individuals. The function *S*_*u*_(*t*|z) is the net survival of the uncured patients, with *S*_*u*_(0|z)=1 and ${\lim }_{t\rightarrow \infty } S_{u}(t|\mathrm {z}) = 0$, and we have that ${\lim }_{t\rightarrow \infty } R(t|\mathrm {z}) = \pi (\mathrm {z})$.

If a net interpretation is not given to the relative survival, *π*(z) can instead be interpreted on an observational level. That is, the population is considered a mixture of cured individuals with the same survival distribution as the general population and uncured individuals with a worse survival described by *S*_*u*_(*t*|z)*S*^∗^(*t*|z). The cure proportion, *π*(z), provides the proportion of individuals with the same survival as the general population [21].

Due to the fact that mainly cured individuals remain alive once *S*_*u*_(*t*|z) becomes sufficiently low, Lambert et al. proposed to compute the cure point as the time at which *S*_*u*_(*t*|z) reaches some MOCR (e.g., 10% or 5%), i.e., *G*(*t*|z)=*S*_*u*_(*t*|z) [9]. This approach was also used by Chauvenet et al. [10]. However, following the argument in “Conditional relative survival” section, it can be difficult to choose a MOCR for *S*_*u*_(*t*|z) if a net survival interpretation is given. If not, *S*_*u*_(*t*|z) remains a relative measure and thus has the same disadvantages as the conditional relative survival approach.

Let *Y* be an unobserved indicator, which is 1 if an individual belongs to the cured group and 0 otherwise. Another cure model-based measure is the conditional probability of cure given survival until time *t*,

4 $$\begin{array}{*{20}l} G(t|\mathrm{z}) &= {\mathrm{P}(Y = 1|T > t, \mathrm{z})} \\ &= \frac{{\mathrm{P}(T > t| Y = 1, \mathrm{z})}{\mathrm{P}(Y = 1|\mathrm{z})}}{{\mathrm{P}(T > t|\mathrm{z})}}\\ &= \frac{S^{*}(t|\mathrm{z})\pi(\mathrm{z})}{S^{*}(t|\mathrm{z})R(t|\mathrm{z})} = \frac{\pi(\mathrm{z})}{R(t|\mathrm{z})},  \end{array} $$

which was proposed for cure point estimation by Boussari et al. and Romain et al. [13, 14]. The cure point was estimated as the time point at which the probability of cure was sufficiently close to one, e.g., exceeding 95%. This measure provides a more intuitive interpretation than *S*_*u*_(*t*), but relies on accurate estimation of *π*(z). Indeed, identifiability is a major issue in cure models, which may lead to highly biased estimates of *π*(z). For a discussion on identifiability of cure models, we refer the reader to the study by Hanin and Huang [22]. Solutions to avoid the issue of identifiability, also applied by Boussari et al. and Romain et al., are available and will be discussed in Section 4.

### Alternative dynamic measures of excess mortality

#### Conditional probability of cancer-related death

The severity of a cancer can be assessed through the cumulative incidence of cancer-related death which can be derived from either cause of death information or relative survival. By using the cumulative incidence, we derive the probability,

5 $$\begin{array}{*{20}l}{} G(t|\mathrm{z}) &= {\mathrm{P}(D = \text{cancer} | T > t, \mathrm{z})} \\ &= \frac{{\mathrm{P}(D = \text{cancer}, T > t| \mathrm{z})}}{{\mathrm{P}(T > t|\mathrm{z})}}\\ &= \frac{{\mathrm{P}(T \!<\! \infty, D =\! \text{cancer}|\mathrm{z})} \,-\, {\mathrm{P}(T \!\leq\! t, D =\! \text{cancer}|\mathrm{z})}}{{\mathrm{P}(T \!>\! t|\mathrm{z})}}\!,  \end{array} $$

where P(*T*<*∞*,*D*=cancer|z)=P(*D*=cancer|z) is the probability of dying due to the cancer given covariates z. The function in (5) is the conditional probability of eventually dying from cancer given survival until time *t*. Since all patients are bound to die at some point, computing 1−P(*D*=cancer|*T*>*t*|z) yields the conditional probability of eventually dying from other causes than cancer. For a given MOCR, the cure point can then be estimated as the time at which the probability of cancer-related death falls below the margin. Although this function provides an understandable measure of the dynamic cancer-related mortality risk, computing P(*D*=cancer|z) generally requires extrapolation beyond the available follow-up. Techniques, often involving parametric survival models, are available to carry out extrapolation but this may lead to biases. To cope with this, Eloranta et al. [16], who originally introduced (5), considered models that ensure P(*D*=cancer)=P(*T*≤*t*_*c*_,*D*=cancer) for a finite time point *t*_*c*_. However, we do not require this restriction for now.

#### Loss of lifetime

Due to its straightforward interpretation, the mean residual lifetime, which can be computed by $\int _{t}^{\infty } S(u|\mathrm {z})du / S(t|\mathrm {z})$, is occasionally used as an alternative to conventional effect measures in survival analysis. In addition to the already introduced measures, we consider the *loss of lifetime* function, given as the difference between the mean residual lifetime of the general population and the patient population, i.e.,

6 $$ G(t|\mathrm{z}) = \frac{\int_{t}^{\infty} S^{*}(u|\mathrm{z})du}{S^{*}(t|\mathrm{z})} - \frac{\int_{t}^{\infty} S(u|\mathrm{z})du}{S(t|\mathrm{z})}.   $$

This function yields the conditional number of years lost due to the cancer given survival until time *t* after the diagnosis. Similarly to the probability of cancer-related death, the cure point can be defined as the time at which the loss of lifetime reaches some MOCR. Also here, the measure generally requires extrapolation beyond the available follow-up. To avoid extrapolation, a restricted loss of lifetime measure could be used, where *∞* in (6) is replaced by some upper limit, *τ*, within the follow-up. However, the interpretation of the restricted loss of lifetime is slightly more awkward as compared to the unrestricted loss of lifetime, which makes it difficult to define a MOCR. Generally, this would depend on the chosen upper limit, *τ*. Therefore, we do not consider the restricted loss of lifetime here.

### Computational considerations

#### Extrapolation

In order to compute the loss of lifetime and probability of cancer-related death from right-censored follow-up data, extrapolation of the involved survival functions and cause-specific hazard functions beyond the available follow-up is required. For the loss of lifetime function, the expected survival, *S*^∗^(*t*|z), can be extrapolated using the method of Ederer et al. [23] (Ederer I) and by making assumptions about the future population mortality rates [15]. For extrapolating the patient survival, *S*(*t*|z), a common strategy is to incorporate external data to provide accurate extrapolations [24]. In particular, parametric relative survival models enable extrapolation by decomposing the patient survival into

7 $$ S(t|\mathrm{z}) = R(t|\mathrm{z}) S^{*}(t|\mathrm{z}).  $$

The Ederer I method enables extrapolation of *S*^∗^, while extrapolation of *R* is possible from any parametric model. Thus, extrapolation is carried out by modelling the relative survival given data with limited follow-up and then making assumptions about how the relative survival continues beyond the follow-up. Based on long term survival data and simulations, a recent assessment of different models for computing the loss of lifetime function did not demonstrate any consistently superior relative survival model [17]. In general, extrapolation beyond the available follow-up is hazardous as even a well-fitting model may extrapolate poorly and the accuracy of the extrapolation cannot be validated.

For the conditional probability of cancer-related death, the cancer-specific cumulative incidence is computed by

8 $$ {\mathrm{P}(T \leq t, D = \text{cancer}|\mathrm{z})} = \int_{0}^{t} S(u|\mathrm{z})\lambda(u|\mathrm{z})du,   $$

where *λ* is the cancer-specific hazard function. Cause of death information is required to compute (8), but these are often incomplete or difficult to determine. Instead of relying on exact cause of death information, parametric relative survival models can be used to compute (8) by making the decomposition *S*(*t*|z)=*S*^∗^(*t*|z)*R*(*t*|z), assuming *λ*(*t*|z)=*h*(*t*|z)−*h*^∗^(*t*|z), and using a parametric model to extrapolate both functions [25]. Again, *S*^∗^(*t*|z) can be extrapolated using the Ederer I method.

The cure model in (3) is typically fitted using simple parametric models, e.g., a Weibull model for *S*_*u*_, such that the relative survival approaches *π*(z) as time approaches infinite [9]. Therefore, if (4) does not reach the chosen MOCR within the follow-up, extrapolation is needed to compute the cure point. This is directly enabled by the parametric cure models.

#### Flexible parametric relative survival models

As a flexible alternative to simple parametric survival models, such as the Weibull or log-normal model, Royston and Parmar introduced a fully parametric proportional hazards model with the log cumulative baseline hazard modelled by restricted cubic splines [26]. This model was extended to relative survival by Nelson et al. who modelled the log cumulative baseline excess hazard by restricted cubic splines [27]. That is, the relative survival is specified by,

9 $$ \log\left[-\log(R(t|\mathrm{z}))\right] = s_{0}(x, \mathrm\gamma) + \mathrm{z}^{T}\beta,   $$

where *s*_0_ is a restricted cubic spline and *x*= log(*t*). We refer to this model as the NRS model.

Andersson et al. altered the base functions of the restricted cubic splines in (9) to establish a cure model, which we will refer to as the ARS model [28]. In this model, the excess hazard is restricted to be zero beyond the last knot of the splines, resulting in a flat relative survival after this point. Thus, the last knot can be considered a *strict* cure point because *h*(*t*|z)−*h*^∗^(*t*|z)=0 beyond this point. The knots of the splines are decided upon by the user prior to the model fitting, which implies that the strict cure point is defined by the user. A common approach is to place the last knot at the last uncensored event time [26, 28], but alternatives can easily be applied. This model is particularly useful for computing (4) and (5) as extrapolation is not needed if the last knot is placed within the follow-up. If *t*_*c*_ is the last knot of the splines, then *π*(z)=*R*(*t*_*c*_|z) and P(*Y*=1|*T*>*t*,z)=1 for all *t*≥*t*_*c*_ in (4) and P(*D*=cancer)=P(*T*≤*t*_*c*_,*D*=cancer) in (5).

Lastly, we consider the flexible mixture cure (FMC) model introduced by Jakobsen et al. [17], where *S*_*u*_(*t*) is modelled by the splines of the Royston-Parmar model, i.e.,

10 $$ R(t|\mathrm{z}) = \pi(\mathrm{z}) + [1 - \pi(\mathrm{z})] \exp(\!{-\exp({s_{0}(x, \mathrm{\gamma}) + \mathrm{z}^{T}\beta}})).  $$

Due to their flexibility, these three models often provide similar relative survival estimates within the follow-up, but differ in their tails, which controls the trajectory beyond the follow-up. The NRS model is linear on the log-log scale beyond the last knot, while the ARS model is constant after the last knot. The splines of the FMC model are also linear beyond the last knot, but the relative survival is bounded downwards by *π*(z). The rationale for introducing the FMC model is its capability to compute (4) without the assumption of a strict cure point within the follow-up. The NRS, ARS, and FMC models can all be used to compute both (6) and (5), but only the cure models, ARS and FMC, can be used to computed (4). All models can be fitted with maximum likelihood.

### Cure point estimation

Assume that $\hat {G}(t)$ is a monotone estimator of the chosen comparison measure, *G*(*t*), based on a parametric survival model. For a chosen MOCR, *ε*, the cure point is estimated by solving the equation

11 $$ \hat{G}(t|\mathrm{z}) = \epsilon,   $$

with respect to *t*. The estimated cure point is denoted $\hat t_{\epsilon }$. The variance of $\hat t_{\epsilon }$ derived by applying the delta method after appropriate smoothing of the general population survival (see Supplementary A for details) is

12 $$ \text{Var}\left[\hat{t}_{\epsilon}\right] \approx \left(\frac{\partial G(t, \mathrm{z})}{\partial t}\vert_{t = \hat t_{\epsilon}}\right)^{-2}\text{Var}\ [\!{G(t, \mathrm{z})}]\vert_{t = \hat t_{\epsilon}},  $$

where ${\text {Var}\left [G(t, \mathrm {z})\right ]}\vert _{t = \hat t_{\epsilon }}$ is the variance of $G(\hat t_{\epsilon }, \mathrm {z})$ without taking into account the uncertainty of $\hat t_{\epsilon }$, i.e., the point-wise variance of *G* evaluated at $\hat t_{\epsilon }$. Note, the variance is inversely proportional to the derivative of *G*(*t*_*ε*_|z), which implies a small variance whenever the comparison measure is steep and a large variance whenever the comparison measure is flat.

We implemented the presented method for estimating cure points and computing their confidence intervals in the R-package cuRe (https://github.com/LasseHjort/cuRe). The package contains functions for fitting the FMC model as well as computing the probability of cure (4), the probability of cancer-related death (5), and the loss of lifetime (6). The comparison measures can be computed using both the NRS, ARS, and the FMC model, except for the probability of cure, which can only be computed from cure models.

## Simulation study

### Simulation design

To illustrate the issue of estimating cure points when based on comparison measures where extrapolation is needed, we conducted a simulation study. Data were simulated according to three relative survival scenarios using a net survival approach, where the time to disease-related death, *T*_*D*_, and the time to death from other causes, *T*_*P*_, are assumed independent [29]. The survival times *T*_*D*_ were simulated from the relative survival, *R*(*t*), and *T*_*P*_ was simulated from the a general population survival function, *S*^∗^(*t*). The observed event time was *T*= min(*T*_*D*_,*T*_*P*_). Introducing censoring, the observed follow-up time was min(*T*,*C*), where *C* is the censoring time, and the status indicator was given by **1**[*T*≤*C*].

The general population survival function, *S*^∗^, was derived using a Danish life table from the Human mortality database [30]. For simplicity, all patients were assumed to be 60-year-old females diagnosed in 1980. The relative survival, *R*(*t*), was simulated from the mixture cure model in (3) using a Weibull distribution for *S*_*u*_. We considered three scenarios with varying cure proportions (see Figure B1 and Table B1 for the relative survival trajectories and parameters, respectively). We selected relatively low cure proportions in the simulations to obtain a large number of events, which is generally advantageous for model fitting. In scenario 1 and 3, the relative survival reaches a plateau early in the follow-up, while the plateau is reached at the end of follow-up in scenario 2. To mimic registry data, the censoring distribution was chosen to be uniform(0, 10). The simulations were repeated 500 times using a sample size of 2,000.

### Simulation results

For a given clinical relevant margin, *ε*, and comparison measure, *G*(*t*), the true cure point, *t*_*ε*_, was obtained by inserting the true mixture cure model into *G*(*t*) and solving (11). Cure point estimates, $\hat t_{\epsilon }$, were obtained by fitting a relative survival model to the simulated data, inserting into *G*(*t*) and solving (11). We considered 4 relative survival models for this purpose:

- The ARS model with knots placed at the 0, 20, 40, 60, 80, 99, and 100 percentiles of the uncensored follow-up times.
- The FMC model with knots placed at the 0, 25, 50, 75, and 100 percentiles of the uncensored follow-up times. The cure proportion was modelled using a logit link function as suggested by Lambert et al. [9].
- The NRS model with knots placed at the 0, 20, 40, 60, 80, and 100 percentiles of the uncensored follow-up times.
- The Weibull mixture cure model from which the data were simulated. This model was included to assess the performance of the “true” model.

The bias of the cure point was computed by the mean absolute deviation, i.e., $\frac {1}{500}\sum _{j = 1}^{500} |\hat t_{\epsilon, j} - t_{\epsilon }$|, where $\hat t_{\epsilon, j}$ is the cure point estimate in the j^th^ simulation. The confidence interval of $\hat t_{\epsilon, j}$ was obtained using (12). The average confidence interval length is reported alongside the cure point bias in Table 2 for each scenario, the four models, and for varying MOCR. The cure point was calculated using the probability of cure in (4), the probability of cancer-related death in (5), and the loss of lifetime in (6).

**Table 2:** Bias and CIL of the cure point estimate in simulated data

			ARS model		FMC model		NRS model		Weibull mixture	
	*ε*	*t*_*ε*_	Bias	$\overline {\text {CIL}}(\hat {t}_{\epsilon })$	Bias	$\overline {\text {CIL}}(\hat {t}_{\epsilon })$	Bias	$\overline {\text {CIL}}(\hat {t}_{\epsilon })$	Bias	$\overline {\text {CIL}}(\hat {t}_{\epsilon })$
Conditional probability of cure										
Scenario 1										
	0.05	3.39	0.67	0.65	0.18	0.87			0.11	0.53
	0.10	2.90	0.42	0.65	0.13	0.61			0.09	0.45
	0.15	2.58	0.27	0.59	0.10	0.49			0.08	0.40
Scenario 2										
	0.05	6.04	1.13	0.61	1.39	4.94			0.42	2.08
	0.10	4.59	0.59	0.70	0.85	3.09			0.31	1.55
	0.15	3.73	0.37	0.71	0.59	2.18			0.25	1.26
Scenario 3										
	0.05	2.17	0.15	0.71	0.44	0.65			0.10	0.48
	0.10	1.63	0.09	0.42	0.13	0.39			0.07	0.36
	0.15	1.27	0.07	0.32	0.08	0.31			0.06	0.29
Conditional probability of cancer-related death										
Scenario 1										
	0.05	3.39	0.66	0.66	0.18	0.86	0.52	1.76	0.11	0.53
	0.10	2.89	0.41	0.65	0.13	0.61	0.30	1.25	0.09	0.44
	0.15	2.57	0.27	0.59	0.10	0.48	0.23	0.97	0.08	0.39
Scenario 2										
	0.05	5.95	1.06	0.62	1.23	4.59	18.95	11.43	0.40	1.99
	0.10	4.52	0.54	0.70	0.77	2.86	11.94	12.48	0.30	1.49
	0.15	3.67	0.34	0.71	0.54	2.00	7.62	11.66	0.24	1.20
Scenario 3										
	0.05	2.17	0.14	0.70	0.19	0.64	0.33	1.09	0.10	0.48
	0.10	1.62	0.09	0.41	0.10	0.38	0.20	0.67	0.07	0.35
	0.15	1.26	0.07	0.31	0.07	0.30	0.15	0.49	0.06	0.29
Loss of lifetime										
Scenario 1										
	1.00	3.34	0.60	0.64	0.16	0.80	0.34	1.47	0.10	0.50
	2.00	2.86	0.36	0.61	0.12	0.57	0.21	1.01	0.09	0.42
	3.00	2.55	0.23	0.55	0.09	0.45	0.16	0.77	0.08	0.37
Scenario 2										
	1.00	5.52	0.79	0.61	0.85	3.43	8.81	7.48	0.33	1.63
	2.00	4.20	0.38	0.67	0.52	2.13	4.67	6.72	0.25	1.22
	3.00	3.42	0.24	0.66	0.36	1.48	2.60	5.17	0.20	0.99
Scenario 3										
	1.00	2.15	0.13	0.64	0.16	0.59	0.22	0.92	0.09	0.46
	2.00	1.62	0.08	0.38	0.08	0.36	0.13	0.55	0.07	0.34
	3.00	1.27	0.06	0.29	0.06	0.29	0.10	0.40	0.06	0.28

The cure point estimates were based on the probabiltiy of cure, the probability of cancer-related death, and the loss of lifetime function. The NRS model was not evaluated for the latter measure since this is not a cure model. ARS: relative survival model by Andersson et al. [28], FMC: flexible mixture cure model by Jakobsen et al. [17], NRS: relative survival model by Nelson et al. [27], CIL: confidence interval length

**Fig. 1:**
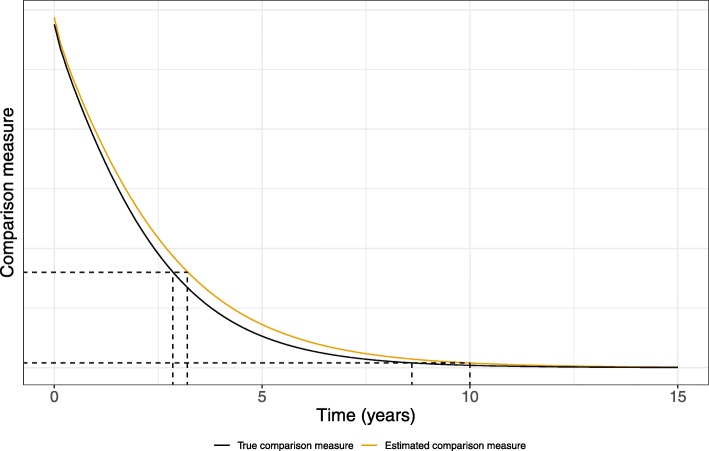
Illustration of how the cure point bias may be larger whenever the comparison measure is flat. Even though the comparison measure is more biased around 3 years compared to 10 years, the cure point bias is larger around 10 years. Thus, in this illustration, selecting a small MOCR leads to higher cure point bias. MOCR: margin of clinical relevance

Overall, the cure point bias seems to decrease with increasing MOCR. This is likely a result of the trajectory of the true comparison measures. As the true comparison measure in each scenario approaches zero, the slope decreases, and the measure flattens out (Figure B.2). This implies that even small differences between the estimated and true comparison measure can yield relatively large biases in the estimated cure point whenever small values of the MOCR are considered (see Fig. 1). This problem is less pronounced for larger MOCRs, as the slope of the true comparison measure also becomes numerically larger at the true cure point.

The true Weibull model yields the most accurate cure point estimates, but provides wide confidence intervals especially in scenario 2, where the relative survival only flattens out by the end of follow-up. More biased cure points were obtained by the FMC and ARS models. Particularly in scenario 2, these models produced cure points biases exceeding half a year. Notably, the FMC model produced wider confidence intervals compared to the ARS models. This can be explained by the identifiability issues associated with the FMC model and the restriction of the ARS model ensuring a constant relative survival beyond its last knot. The former may also explain why a confidence interval could not be computed in 1/500 simulations.

Because the NRS is not a cure model, it could not be used to estimate the cure point based on the conditional probability of cure. The cure point bias of the NRS model based on the two remaining comparison measures was slightly higher compared to the true model in scenario 1 and 3. However, in scenario 2, the bias was extremely large. This is likely due to the extrapolation needed to compute these two comparison measures. The data were simulated from a Weibull mixture cure model, which only becomes flat by the end of the follow-up. When data points are drawn, which introduces random variation, the NRS model may fail to capture that the true relative survival contains a plateau, which yields biased extrapolations.

To conclude, the NRS model seems to provide too biased results, while the true Weibull model will often be too simple in practice. The use of either the ARS or FMC model is likely the best choice for cure point estimation, but large confidence intervals may occur with the FMC model.

## Analysis of danish cancer registry data

### Data description

To exemplify the application of the considered approach for cure point estimation, we analyzed patient data from colon cancer patients retrieved from the Danish Colorectal Cancer Group Database [31]. The registry ensures accurate follow-up on deaths by merging with the Danish Civil Registration System [32]. The selection of colon cancer was based on a previous study displaying statistical cure in patients ≥20 years of age [12]. All patients ≥20 years of age diagnosed between 2000 and 2016 were included. Follow-up was measured as the time from diagnosis until death or censoring (June 2017). Danish general population mortality rates were retrieved from the Human Mortality Database [30]. When mortality rates for calendar years beyond those available in the life table were needed, the age- and sex-specific rates from the last available calendar year were used. The study was approved by the Danish Data Protection Agency (2008-58-0028). For illustrative purposes, we only consider the FMC model here. The FMC model was fitted using four knots placed at the 0, 33, 67, and 100 percentiles of the uncensored follow-up times and a logit link function for the cure proportion.

### Results

**Fig. 2:**
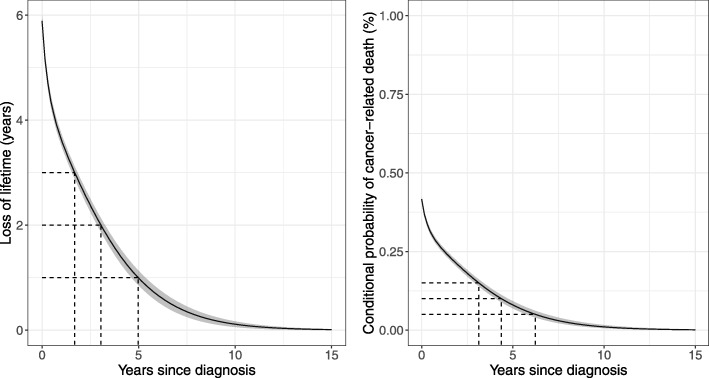
Left: The loss of lifetime function within the first 15 years after diagnosis for Danish colon cancer patients. The dashed lines indicate cure point estimates based on three different clinical relevant margins; 1, 2, and 3 years. Right: The conditional probability of cancer-related death within the first 15 years after diagnosis for Danish colon cancer patients. The clinical relevant margins are 0.05, 0.10, and 0.15. The shaded areas indicate pointwise 95% confidence intervals

In total, 42,380 colon cancer patients were included in the study and the 5-year Kaplan-Meier estimate was 49% (95% CI, 48-49%). The fitted FMC model is shown in Figure B3 together with a non-parametric relative survival estimate calculated by the Ederer I method [23]. Estimates of the 5-year relative survival, the cure proportion, the probability of dying due to cancer, and the baseline (at time zero) loss of lifetime are shown in Table 3. The relative survival had an immediate steep decrease which flattened over time suggesting statistical cure. The loss of lifetime function and the conditional probability of cancer-related death (Fig. 2) demonstrated a similar pattern with both approaching zero as time progresses.

**Table 3:** Relative survival estimates, cure proportion, probability of dying due to cancer, and baseline loss of lifetime estimates for Danish colon cancer patients. RS: relative survival

	n = 42,380
Mean age (range)	72(22-105)
5-year RS (Ederer II)	0.61(0.60-0.61)
5-year RS (parametric)	0.60(0.59-0.61)
Cure proportion	0.54(0.53-0.56)
Probability of dying due to cancer	0.42(0.41-0.42)
Baseline loss of lifetime (years)	5.89(5.79-5.99)

**Fig. 3:**
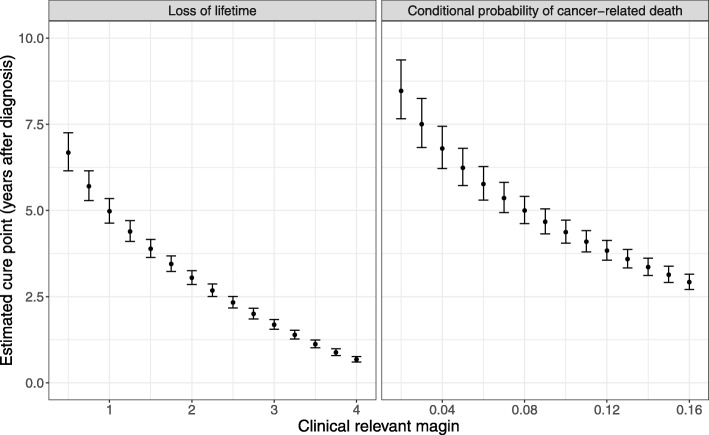
The estimated cure points with 95% confidence intervals against the clinical relevant margin in Danish colon cancer patients. For the loss of lifetime function, the margin is given in years

Figure 3 displays the estimated cure point for the colon cancer patients, obtained by solving (11), as function of the chosen MOCR. Whenever the MOCR is low, small changes to the margin implied substantial changes in the estimated cure point, but for larger values, the cure point was less sensitive towards the choice of margin. For example, increasing the margin for the loss of lifetime function from 3 years to 4 years resulted in a decrease of the cure point estimate from 1.7 to 0.7 years, while increasing the margin from 0.5 to 1.5 years resulted in a decrease from 6.7 to 3.9 years.

**Fig. 4:**
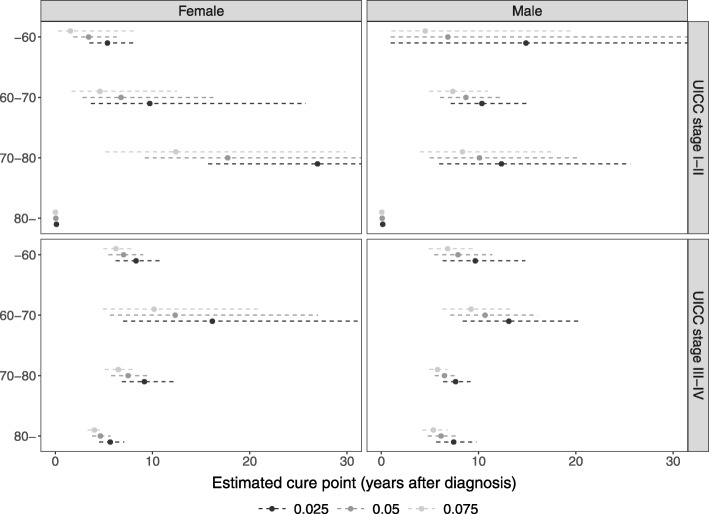
The estimated cure point for Danish colon cancer patients stratified on age group (-60, 60-70, 70-80, 80-), gender, and stage (UICC stage I-II vs. III-IV). The estimates are based on the conditional probability of cancer-related death (see Figures B.4 and B.5). UICC: Union for International Cancer Control

The patients were further stratified according to age group (-60, 60-70, 70-80, 80-), gender, and clinical stage (Union for International Cancer Control stage I-II, III-IV) and the FMC model was fitted to each subgroup separately. The stratified cure point estimates computed with the conditional probability of cancer-related death (see Figures B.4 an B.5) as comparison measure and three MOCRs (0.025, 0.05, and 0.075) are shown in Fig. 4. The cure point estimates of female and male patients displayed similar trends. In certain strata, the confidence interval of the cure point was wide, which makes the usability of these estimates difficult. For other subgroups, the variance was reasonably small and led to stable estimates. The cure point for low stage patients >80 years of age was very small, and due to the slope of the conditional probability of cancer-related death (Figures B.4 an B.5), the corresponding confidence interval was narrow.

## Discussion

Cure points enable communication of prognostic information to cancer patients and are particularly useful for patients attending routine follow-up. Also health care planners may find cure points useful, e.g., for deciding the duration of post-treatment follow-up programs. If an early cure point is detected, the duration may be adjusted accordingly, which potentially lowers the cost of the follow-up program and avoids unnecessary patient anxiety related to routine follow-up. However, the detection of long term toxicities is typically also a goal of routine follow-up programs and should be considered alongside the mortality risk. The search for surrogate endpoints to be used in clinical trials in order to increase the pace at which these are executed has been the focus of recent cancer studies [33, 34]. Cure points may be used to derive new surrogate endpoints, since prolonging the study period beyond the cure point may not be necessary. However, long term risks cannot be observed in studies with short follow-up and additional validation of new endpoints in a series of clinical trials is required before they can be applied in practice [35].

In this article, we have reviewed approaches to estimate cure points from cancer survival data, discussed their usability, and introduced two new useful measures for cure point estimation, namely the conditional probability of cancer-related death and the loss of lifetime function. In recent studies of lymphoma, Maurer et al. and Hapgood et al. used sequential testing of standardized mortality ratios to evaluate the survival improvement of the patients [6, 7]. However, as previously mentioned, approaches that directly involve hypothesis testing are not appropriate for cure point estimation. In our previous work, we combined the same standardized mortality ratio approach with restricted loss of lifetime estimates within lymphoma and concluded that the patients had a sufficiently low restricted loss of lifetime after 2 years of event-free survival [8]. Dal Maso et al. [11] computed the cure point of various cancers using the conditional relative survival approach, while Chauvenet et al. [10] computed the cure point of colorectal cancer patients as the time at which the relative survival of the uncured fell below 10%. Andersson et al. used the loss of lifetime approach to evaluate the survival progression of colon cancer patients and argued that the loss of lifetime was sufficiently low after 8-10 years of survival [12].

In the simulations, the bias of the cure point was computed for three different cure models, namely the FMC, ARS, and the Weibull mixture cure model. The ARS incorporates a strict cure point by assuming a constant relative survival beyond the last knot of the splines [28]. However, estimating the cure point from a model where the strict cure point is explicitly defined seems counter-intuitive and the choice of strict cure point will likely have a large influence on the final cure point estimate. Therefore, this approach may not be ideal for the purpose of estimating cure points and we suggest that other models are used or that the last knot of the ARS model is placed far beyond the point at which the excess hazard can be assumed to be zero. Nevertheless, Boussari et al. applied this model to estimate the cure point through the probability of cure in (4) [13]. As demonstrated in the simulation study, (4) could also be computed from regular cure models where cure occurs at an asymptote (the FMC or the Weibull mixture cure model). However, computing (4) would require reliable cure proportion estimates which can be problematic in some scenarios due to identifiability issues [21].

The main issue associated with the loss of lifetime function and the conditional probability of cancer-related death is the need for extrapolation for which the accuracy cannot generally be quantified. This, combined with the stochastic nature of time to event data, may lead to biased conclusions. As no single extrapolation method is universally superior [17], it is recommended to accompany the cure point estimates with a sensitivity analysis in which different techniques are used if these comparison measures are used.

Deciding on a MOCR is an essential part of the present methodology. This decision is similar to the choice of a non-inferiority margin as done in non-inferiority studies [36]. The choice of MOCR may be based on, e.g., the age and gender distribution of the considered patient population, or if single-patient cure points are of interest, the specific characteristics of the patient. Therefore, it is important that researchers with experience within the field of research aid in deciding on a MOCR which also emphasizes the need for considering comparison measures that are interpretable. It is generally difficult to choose a comparison measure, an estimation procedure for the comparison measure, and a MOCR such that both interpretability and computability are preserved.

In addition, the sensitivity of the cure point towards the choice of MOCR is a major issue. When the applied comparison measure is steep, the cure point is fairly robust against small changes in the chosen MOCR, whereas the cure point becomes very sensitive to small changes in the MOCR whenever the comparison measure is flat. Additionally, the variance of the cure point estimator is inversely proportional to the derivative of the comparison measure evaluated at the estimated cure point. This may lead to very large confidence intervals if the slope of the comparison measure is small at the estimated cure point, making the cure point estimate difficult to apply in practice. Thus, computing cure points is a challenging task and we suggest that extensive sensitivity analyses are conducted if cure point estimates are of interest.

It is worthwhile to pay attention to the interpretation of the cure point. If the patient mortality risk reaches the same level as the general population mortality risk, it may be tempting to conclude that the patients have the expected survival they would have experienced had they never had cancer. While this is likely of interest to the patients, this conclusion cannot be drawn from these analyses as the general population is typically only matched on variables such as age, gender, and calendar year. If a study includes patients treated with a specific therapy, which is typically not given to frail patients, or merely includes patients with high socioeconomic status, the resulting patient population may be in slightly better shape than the general population. Thus, for patients achieving statistical cure, a significant excess mortality risk may still exist if compared to non-diseased individuals with similar physical capabilities or similar socioeconomic status. Information on physical condition and socioeconomic status is typically not included in publicly available life tables and thus cannot readily be taken into account.

Because of the varying survival trajectory of cancers, cure points are not useful for all diseases, e.g., cancers where statistical cure is not achieved such as bladder cancer [15]. To accompany analyses of statistical cure, a formal test for the assumption of statistical cure would be convenient. Formal tests based on simple parametric models have previously been introduced [37, 38], but these are not commonly used and suffer from a number of practical disadvantages [39].

## Conclusion

Cure point estimation is challenging and requires careful selection of an interpretable measure which quantifies the mortality difference between the patient and general population, a MOCR, and an estimation procedure that can accurately estimate the dynamic mortality difference. Additionally, the cure point may be sensitive to small changes in the MOCR while large confidence intervals may be obtained dependent on the behavior of the comparison measure. Therefore, if cure points are of interest, sensitivity analysis are encouraged and the limitations of the applied approach should be clearly stated.

## Supplementary information

**Additional file 1** Supplementary material.

## Data Availability

Data used to generate the findings of the study were obtained from the Danish Clinical Registries (Danish Colorectal Cancer Group Database) after approval of our study plan by the registry and the Danish Data Protection Agency. The registry contains patient identifiable information and therefore sharing of these data is not allowed per the terms of the agreement with the registry. However, from the registry, access to the data is granted on a case to case basis after submission and approval of an appropriate study plan and reasonable data request. Data from the Danish Clinical Registries can be applied for at http://www.rkkp.dk/forskning. The code used for generating the results can be found at https://github.com/LasseHjort/CurePointEstimation.

## Abbreviations

ARS: Andersson et al. [28] relative survival
FMC: Flexible mixture cure
MOCR: Margin of clinical relevance
NRS: Nelson et al. [27] relative survival
