# Supplementary material for: On estimating the time to statistical cure
Source: BMC Med Res Methodol. 2020 Mar 26;20:71. doi: 10.1186/s12874-020-00946-8 (PMC7098130; doi:10.1186/s12874-020-00946-8)
Supplement: Supplementary file 1 — Additional file 1 Supplementary material. [file 12874_2020_946_MOESM1_ESM.pdf]

Appendix A: Variance of the cure point estimator

In the following, we derive a variance estimator for the cure point estimator using the delta method under the assumption that the hazard function $h(t, \boldsymbol{\theta})$ has a parametric form with parameters $\boldsymbol{\theta}$. Denote the parameter estimate by $\hat{\boldsymbol{\theta}}$, and assume that $\sqrt{n}(\hat{\boldsymbol{\theta}} - \boldsymbol{\theta}_0)$ is asymptotically normal with mean $\mathbf{0}$ and variance Σ, where $\boldsymbol{\theta}_0$ is the true parameter value and Σ is the inverse information matrix, i.e., minus the inverse of the expected Hessian matrix of the likelihood function evaluated at $\boldsymbol{\theta}_0$.

Let $G(t, \boldsymbol{\theta}) = G(h, h^*)(t)$ be the strictly monotone comparison measure at time t obtained by inserting the parameters of the hazard function into the comparison measure and assume that G is continuously differentiable with respect to $\boldsymbol{\theta}$ and t. Furthermore, let $t_\epsilon = G^{-1}(\epsilon, \boldsymbol{\theta})$ and $\hat{t}_\epsilon = G^{-1}(\epsilon, \hat{\boldsymbol{\theta}})$ for a fixed clinical relevant margin, ϵ. The variance of $\hat{t}_\epsilon$ can then be approximated directly by using the the delta method, i.e.,

$$\mathrm{Var}\left[\hat{t}_\epsilon\right] \approx \frac{1}{n}\left(\nabla_\theta t_\epsilon|_{\boldsymbol{\theta}=\hat{\boldsymbol{\theta}}}\right)\Sigma\left(\nabla_\theta t_\epsilon|_{\boldsymbol{\theta}=\hat{\boldsymbol{\theta}}}\right)^T. \tag{1}$$

Due to the definition of t_ϵ,

$$\nabla_{\boldsymbol{\theta}} G(t_\epsilon, \boldsymbol{\theta})|_{\boldsymbol{\theta}=\hat{\boldsymbol{\theta}}} = \mathbf{0}, \tag{2}$$

and by the chain rule of vector functions we have that

$$\nabla_{\boldsymbol{\theta}} G(t_\epsilon, \boldsymbol{\theta})|_{\boldsymbol{\theta}=\hat{\boldsymbol{\theta}}} = \frac{\partial G(t, \boldsymbol{\theta})}{\partial t}|_{t=\hat{t}_\epsilon, \boldsymbol{\theta}=\hat{\boldsymbol{\theta}}} \nabla_\theta t_\epsilon|_{\boldsymbol{\theta}=\hat{\boldsymbol{\theta}}} + \nabla_{\boldsymbol{\theta}} G(t, \boldsymbol{\theta})|_{t=\hat{t}_\epsilon, \boldsymbol{\theta}=\hat{\boldsymbol{\theta}}}.$$

Thus,

$$\nabla_\theta t_\epsilon|_{\boldsymbol{\theta}=\hat{\boldsymbol{\theta}}} = -\left(\frac{\partial G(t, \boldsymbol{\theta})}{\partial t}|_{t=\hat{t}_\epsilon, \boldsymbol{\theta}=\hat{\boldsymbol{\theta}}}\right)^{-1} \nabla_{\boldsymbol{\theta}} G(t, \boldsymbol{\theta})|_{t=\hat{t}_\epsilon, \boldsymbol{\theta}=\hat{\boldsymbol{\theta}}}. \tag{3}$$

Inserting into (1) yields

$$\mathrm{Var}\left[\hat{t}_\epsilon\right] \approx \frac{1}{n}\left(\frac{\partial G(t, \boldsymbol{\theta})}{\partial t}|_{t=\hat{t}_\epsilon, \boldsymbol{\theta}=\hat{\boldsymbol{\theta}}}\right)^{-2}\left(\nabla_{\boldsymbol{\theta}} G(t, \boldsymbol{\theta})|_{t=\hat{t}_\epsilon, \boldsymbol{\theta}=\hat{\boldsymbol{\theta}}}\right)\Sigma\left(\nabla_{\boldsymbol{\theta}} G(t, \boldsymbol{\theta})|_{t=\hat{t}_\epsilon, \boldsymbol{\theta}=\hat{\boldsymbol{\theta}}}\right)^T \tag{4}$$

$$\approx \left(\frac{\partial G(t, \boldsymbol{\theta})}{\partial t}|_{t=\hat{t}_\epsilon, \boldsymbol{\theta}=\hat{\boldsymbol{\theta}}}\right)^{-2} \mathrm{Var}\left[G(t, \hat{\boldsymbol{\theta}})\right]|_{t=\hat{t}_\epsilon}, \tag{5}$$

where $\mathrm{Var}\left[G(t, \hat{\boldsymbol{\theta}})\right]|_{t=\hat{t}_\epsilon}$ is the variance of $G(\hat{t}_\epsilon, \hat{\boldsymbol{\theta}})$ without taking into account the uncertainty of $\hat{t}_\epsilon$, i.e., the point-wise variance of G evaluated at the point $\hat{t}_\epsilon$. For obtaining a non-negative confidence interval for the cure point, $\hat{t}_\epsilon$, the variance of the log-transformed estimator is computed by the delta method:

$$\mathrm{Var}\left[\log(\hat{t}_\epsilon)\right] \approx \frac{1}{\hat{t}_\epsilon^2}\mathrm{Var}\left[\hat{t}_\epsilon\right] \approx \frac{1}{\hat{t}_\epsilon^2}\left(\frac{\partial G(t, \boldsymbol{\theta})}{\partial t}|_{t=\hat{t}_\epsilon, \boldsymbol{\theta}=\hat{\boldsymbol{\theta}}}\right)^{-2} \mathrm{Var}\left[G(t, \hat{\boldsymbol{\theta}})\right]|_{t=\hat{t}_\epsilon}. \tag{6}$$

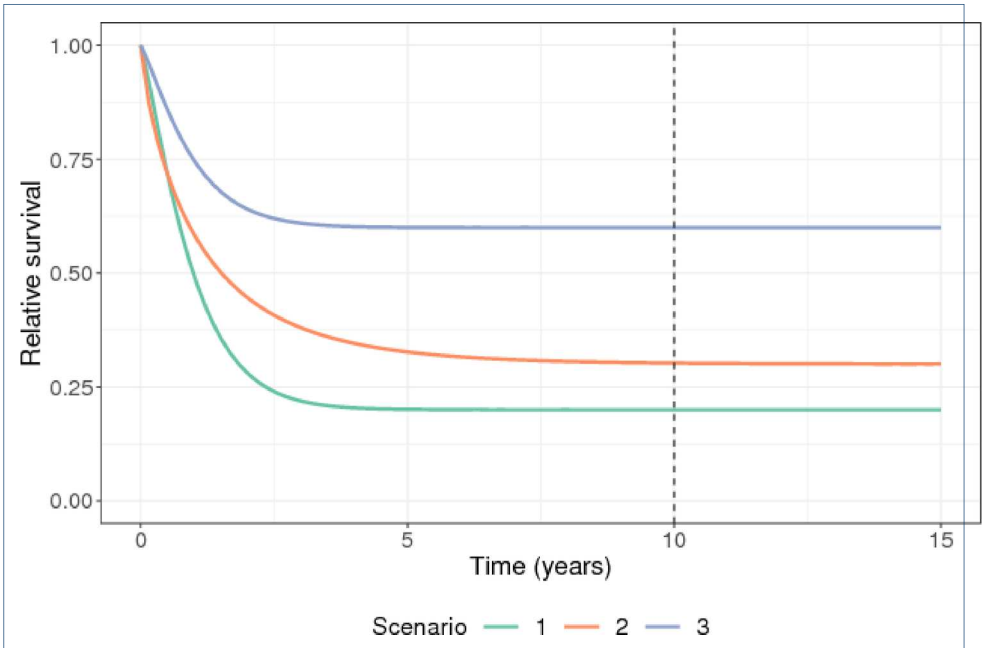

Figure B.1 The relative survival trajectories of the models from which the simulated data were drawn.

Appendix B: Additional figures and tables

Data were simulated from a Weibull mixture cure model, formulated by

$$R(t) = \pi + (1 - \pi)\exp\left(\gamma_2 t_1^{\gamma}\right), \tag{7}$$

with parameter values displayed in Table B.1.

Scenario	π	γ_1	γ_2
1	0.2	1.2	1
2	0.3	0.8	0.9
3	0.6	1.2	1

Table B.1 Parameter values used for simulating survival data.

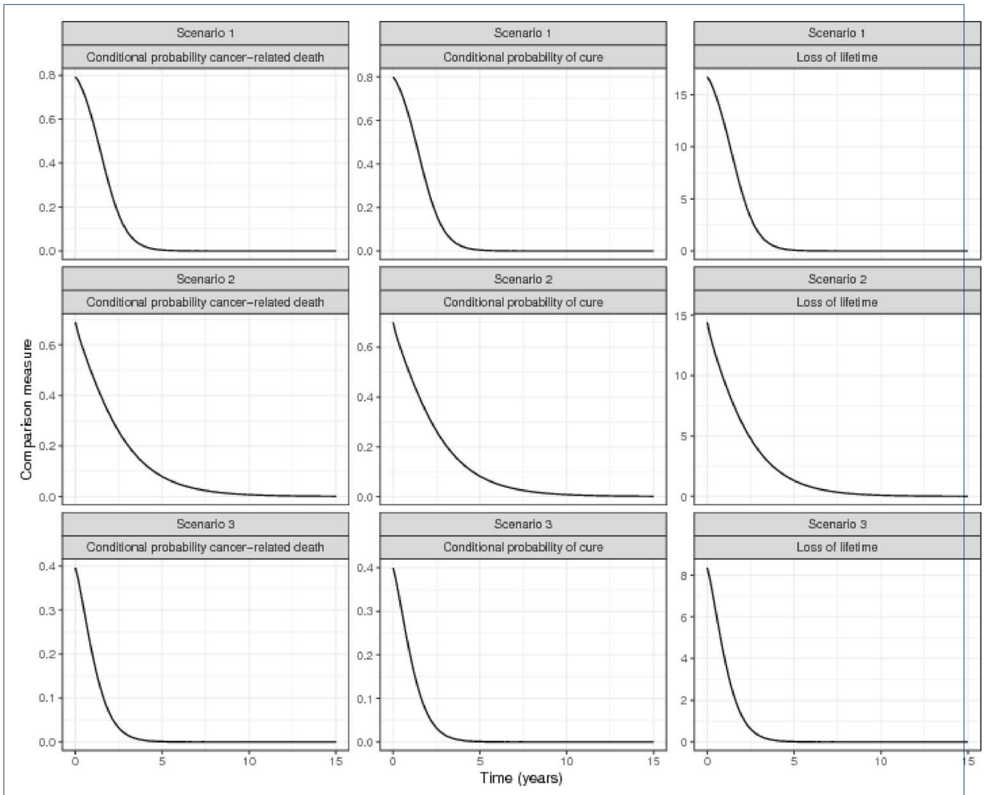

Figure B.2 The true trajectory of the conditional probability of cure, conditional probability cancer-related death, and loss of lifetime function obtained by inserting the true relative survival function in the formula for each comparison measure. The true relative survival for each scenario can be found in Figure B.1.

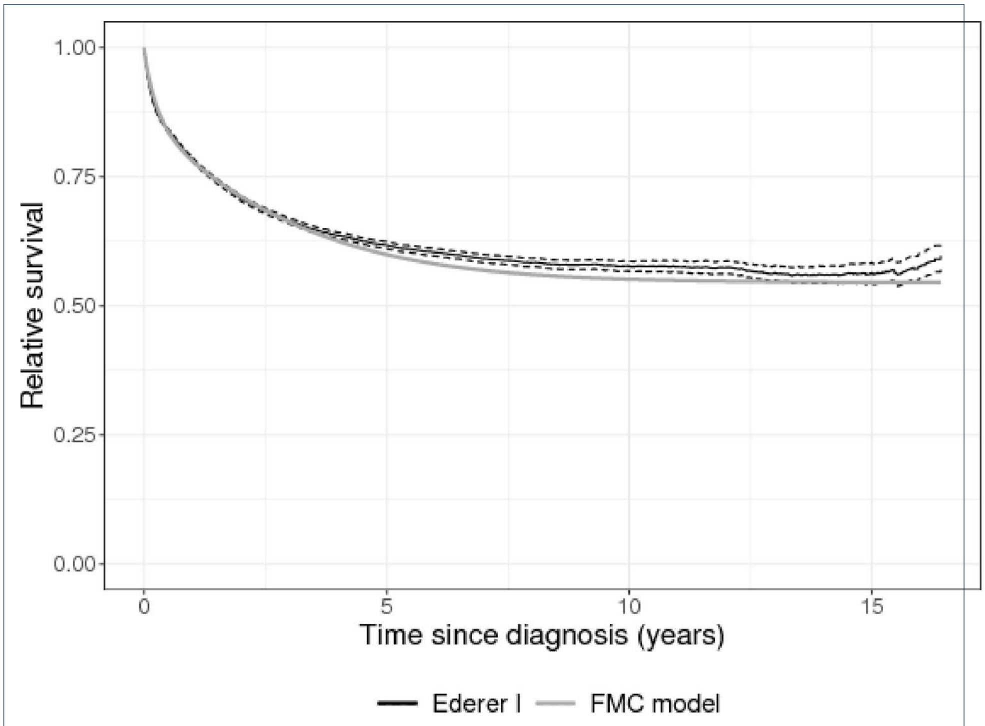

Figure B.3 The relative survival of Danish colon cancer patients calculated by the Ederer I method including 95% confidence intervals (dashed lines) and the FMC model. FMC: flexible mixture cure.

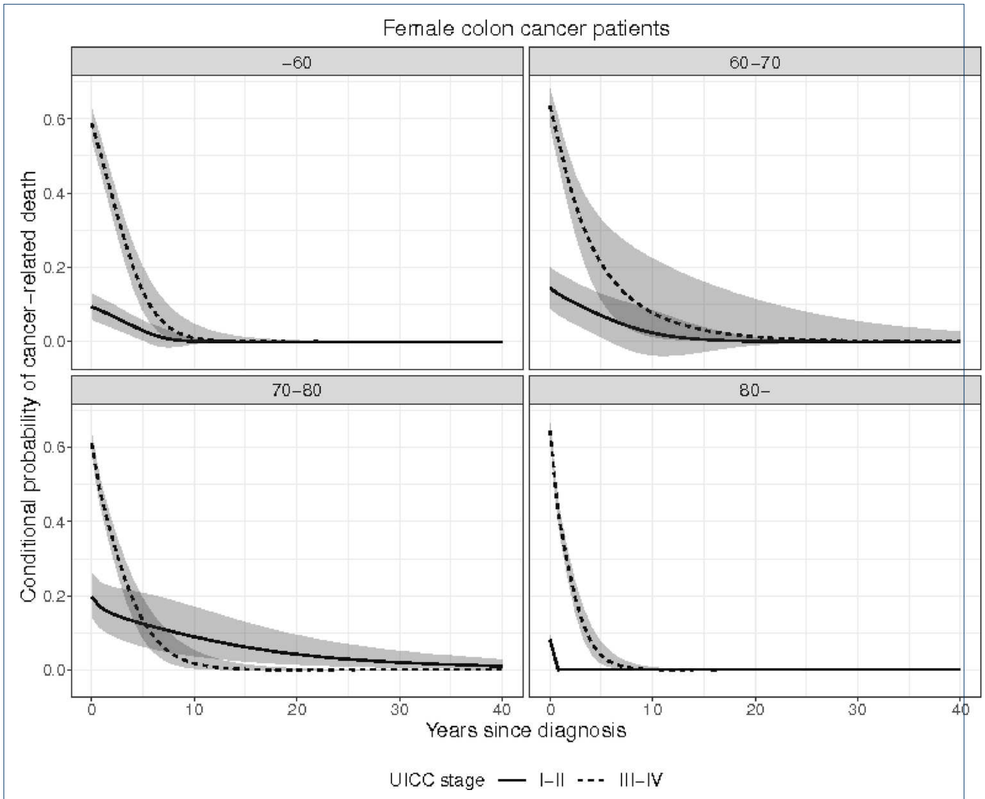

Figure B.4 The conditional probability of cancer-related death in Danish female colon cancer patients stratified on age group (-60, 60-70, 70-80, 80-) and stage (UICC stage I-II vs III-IV). UICC: Union for International Cancer Control.

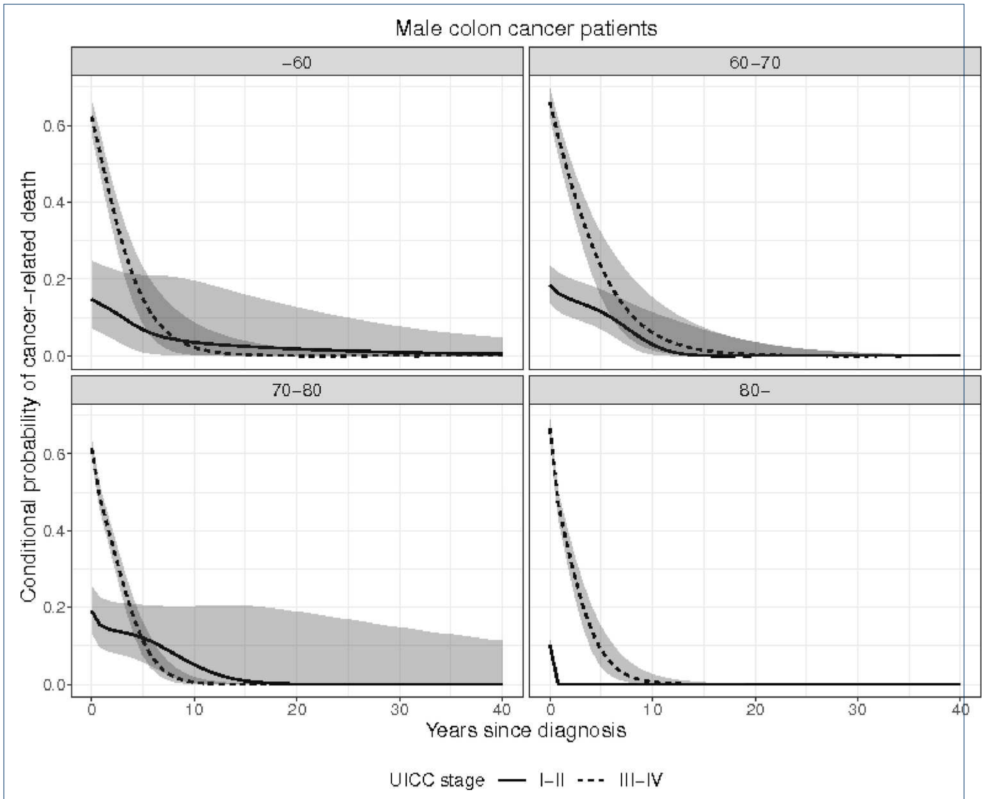

Figure B.5 The conditional probability of cancer-related death in Danish male colon cancer patients stratified on age group (-60, 60-70, 70-80, 80-) and stage (UICC stage I-II vs III-IV). UICC: Union for International Cancer Control.
